# Lung adenocarcinoma and colorectal cancer as double primary malignancies reveal lynch syndrome: a case report of germline MLH1 mutation with response to immunotherapy and familial aggregation

**DOI:** 10.3389/fimmu.2025.1709036

**Published:** 2026-01-14

**Authors:** Menglei Wang, Haoyue Xue, Jiamin Hong, Qi Liu, Keke Hu, Chang Shu, Jinghua Wang, Yu Long, Yehong Han, Xuefei Yang, Jinhua Lu

**Affiliations:** 1 School of Basic Medical Sciences, Zhejiang Chinese Medical University, Hangzhou, China; 2 Department of Oncology, Hangzhou TCM Hospital of Zhejiang Chinese Medical University, Hangzhou Hospital of Traditional Chinese Medicine, Hangzhou, China; 3 Department of Pathology, Hangzhou TCM Hospital of Zhejiang Chinese Medical University, Hangzhou Hospital of Traditional Chinese Medicine, Hangzhou, China; 4 Department of Gastrointestinal Surgery, Hangzhou TCM Hospital of Zhejiang Chinese Medical University, Hangzhou Hospital of Traditional Chinese Medicine, Hangzhou, China

**Keywords:** dMMR, immunotherapy, lynch syndrome, MLH1, retrospective MMR testing

## Abstract

Lynch syndrome (LS), also known as hereditary nonpolyposis colorectal cancer, is a genetic condition that increases the risk of developing colorectal cancer (CRC) and other cancers due to defective DNA mismatch repair (dMMR). This article reports a case of a patient who developed lung adenocarcinoma followed by CRC. The detection of dMMR by immunohistochemistry in both the metastatic lesion and CRC led to retrospective testing, which revealed a concomitant loss of MLH1 and PMS2 in the primary lung cancer. Germline testing subsequently confirmed a diagnosis of LS associated with an MLH1 mutation, with significant familial clustering observed. The patient responded effectively to anti-PD-1 immunotherapy. This case highlights that lung adenocarcinoma can be a manifestation of LS and underscores the critical importance of retrospective MMR testing in establishing the diagnosis. Furthermore, it demonstrates the efficacy of immune checkpoint inhibitions in advanced dMMR tumors.

## Introduction

Lynch syndrome (LS) is an autosomal dominant hereditary disorder caused by defective DNA mismatch repair (dMMR), primarily characterized by familial clustering of colorectal cancer (CRC), endometrial cancer, and other associated malignancies (1). MLH1, MSH2, MSH6, and PMS2 are the principal mismatch repair genes in which mutations most frequently occur (2). dMMR results in microsatellite instability-high (MSI-H) status due to accumulation of errors in short tandem repeats. Lung adenocarcinoma is relatively rare as an LS-associated tumor, with an incidence of only 0.1% to 1.0% (3). Therefore, LS is often overlooked when initially manifesting as or coinciding with non-core tumors, such as lung cancer. The elevated tumor mutational burden characteristic of dMMR/MSI-H tumors generates abundant neoantigens, rendering these cancers susceptible to PD-1/PD-L1 inhibitors (4). Here, we report a case of LS manifesting as dual primary lung and colorectal cancers, confirmed via retrospective MMR testing and germline genetic analysis, with a robust response to immunotherapy and identification of a germline MLH1 mutation with familial segregation.

## Case presentation

A 59-year-old non-smoking female presented with a two-year history of recurrent right-sided chest pain and underwent computed tomography (CT) on 21 March 2023, which revealed a mass in the right middle lobe suggestive of malignancy. She underwent resection one week later. Pathological examination confirmed a poorly differentiated invasive adenocarcinoma (3.2 × 2.8 × 1.4 cm) with extensive necrosis and visceral pleural invasion. Surgical margins were negative. Lymph node metastases were present in station 7 (3/3 positive) and absent in station 10 (0/1). Immunohistochemistry (IHC) results were as follows: TTF-1 (+), Napsin A (+), P63 (-), P40 (-), CK5/6 (-), CgA (-), Syn (partial+), Ki-67 (+), P53 (strongly+ in most cells), EGFR (weakly+ in a subset), Vimentin (-), and CD34 highlighting vascular tumor emboli. Molecular profiling identified mutations in KRAS and PTEN, with PD-L1 TPS = 15%. The patient received four cycles of pemetrexed/carboplatin chemotherapy concurrent with radiotherapy starting on 6 May 2023, followed by regular surveillance.

**Figure 1:**
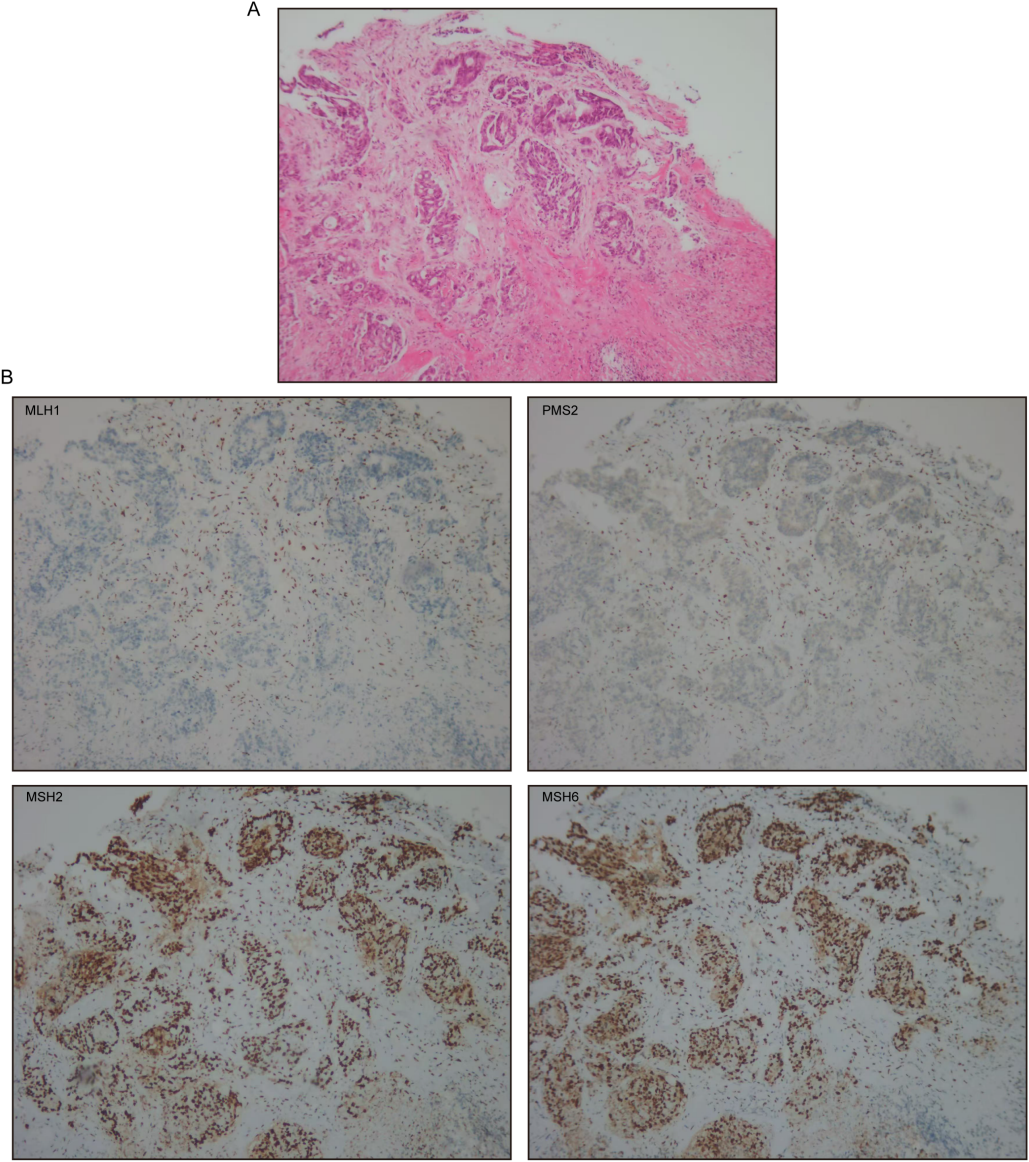
Colorectal cancer. **(A)** H&E staining at ×100. **(B)** MLH1, PMS2, MSH2, and MSH6 immunohistochemical stain at ×100.

**Figure 2:**
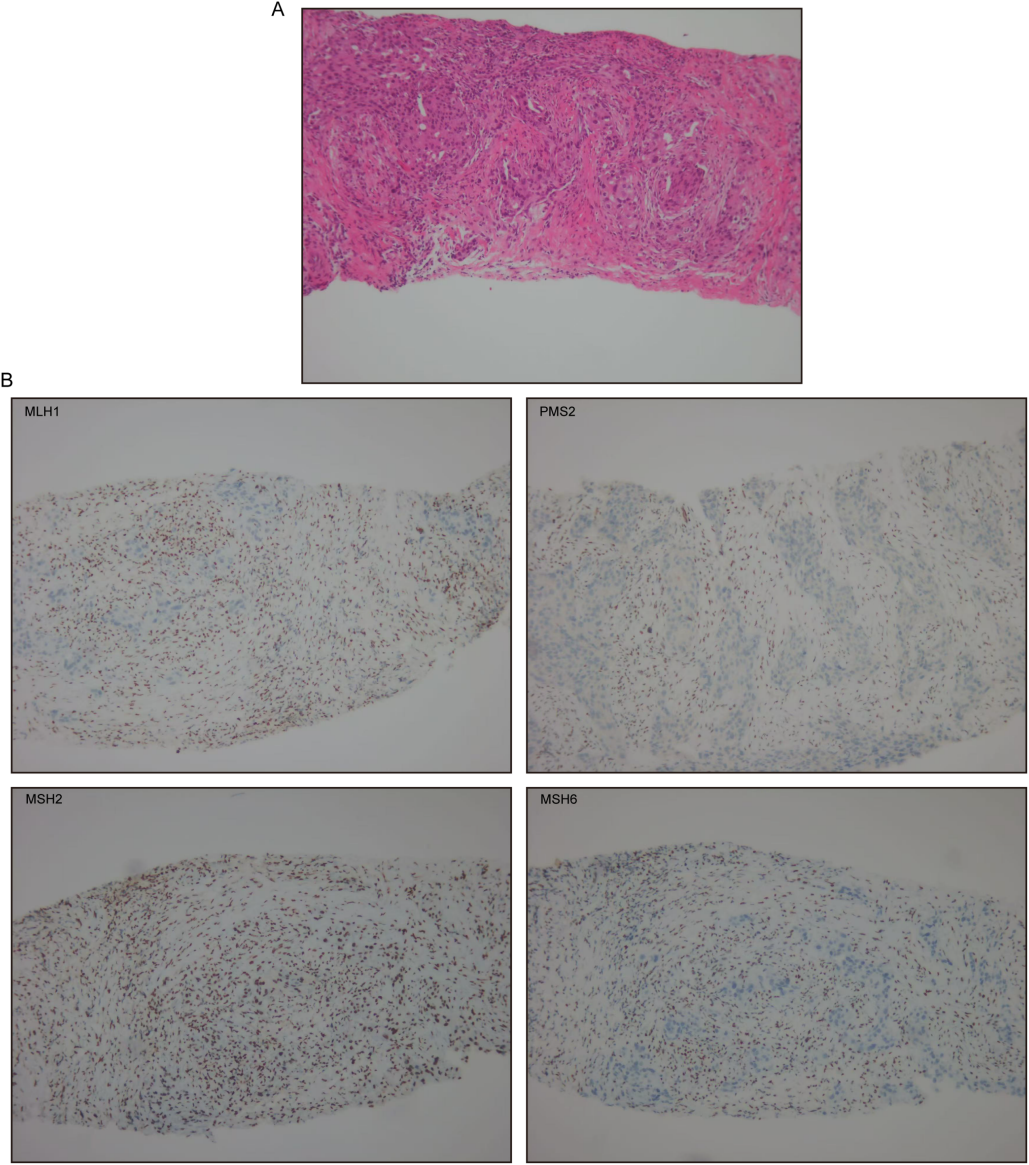
Metastatic lung adenocarcinoma. **(A)** H&E staining at ×100. **(B)** MLH1, PMS2, MSH2, and MSH6 immunohistochemical stain at ×100.

In March 2025, follow-up evaluations identified a rectal mass and a right paraspinal lesion. Colonoscopy identified a cecal adenocarcinoma and ascending colon tubular-villous adenoma with high-grade dysplasia evolving to moderately differentiated mucosal carcinoma. Abdominal CT demonstrated rectal tumor infiltration of perirectal fat with regional lymphadenopathy. IHC of the rectal tumor showed loss of MLH1 and PMS2, with intact MSH2 and MSH6 (**Figure 1**). Biopsy of the paraspinal mass confirmed metastatic lung adenocarcinoma with loss of MLH1, PMS2, and MSH6, and retained MSH2 expression (**Figure 2**).

**Figure 3:**
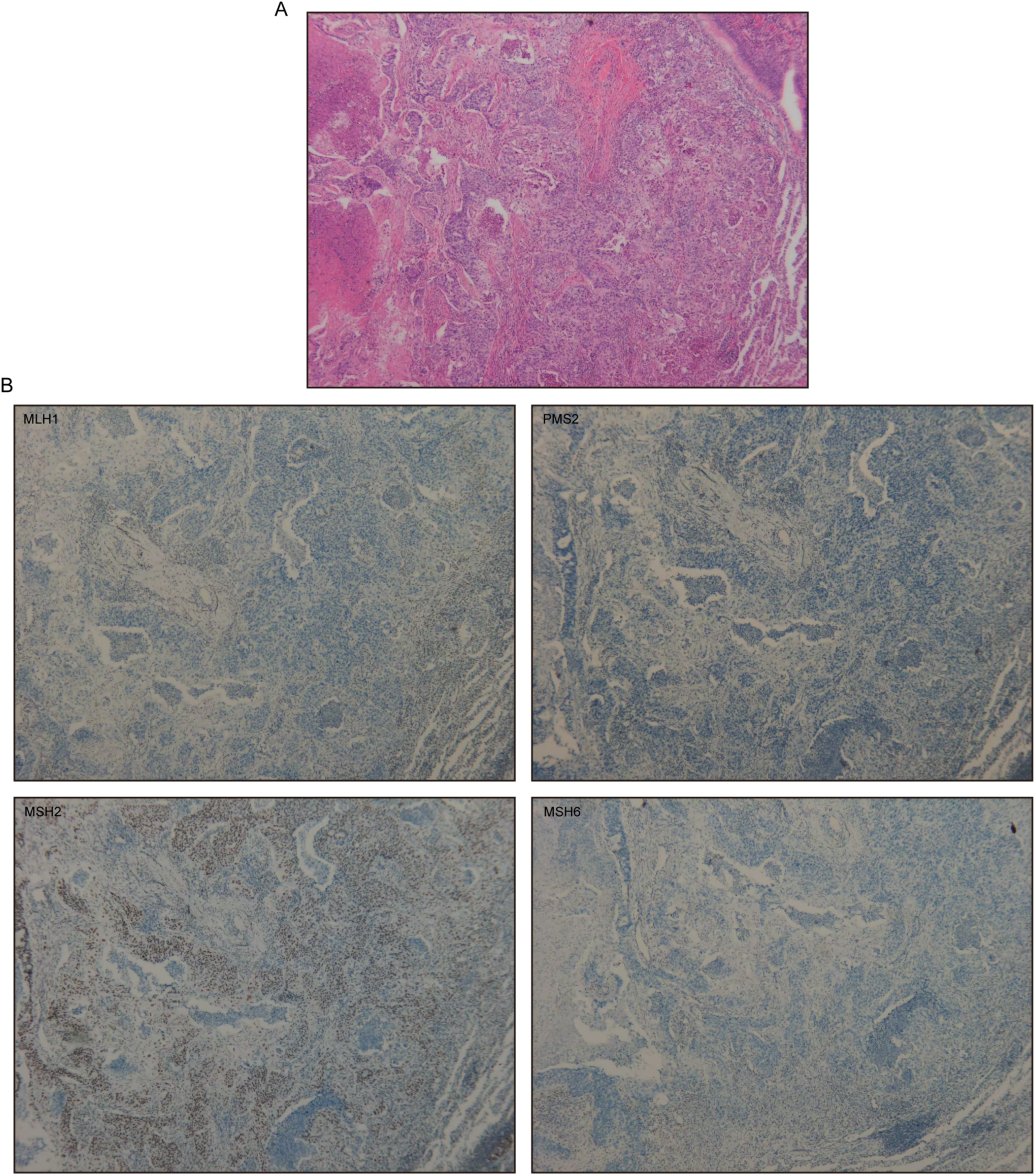
Primary lung adenocarcinoma. **(A)** H&E staining at ×100. **(B)** MLH1, PMS2, MSH2, and MSH6 immunohistochemical stain at ×100.

Given discordant MMR protein expression in the metastasis, the primary lung cancer paraffin block was reanalyzed, confirming concordant dMMR (MLH1/PMS2 deficient) with CRC and metastatic site (**Figure 3**). These findings strongly suggested LS. Germline genetic testing performed in July 2025 for the patient, her son, and her daughter identified a heterozygous frameshift mutation in exon 13 of MLH1 (p.P496Afs*10) in all three individuals. Subsequently, the 37-year-old daughter was diagnosed with sigmoid colon adenocarcinoma via a colonoscopy biopsy, and MMR testing similarly indicated MLH1/PMS2 deficiency. Furthermore, family history review confirmed that the patient’s older brother had been diagnosed with colorectal cancer before age 50. Based on the revised Bethesda guidelines (5), a clinical diagnosis of LS was established.

**Figure 4:**
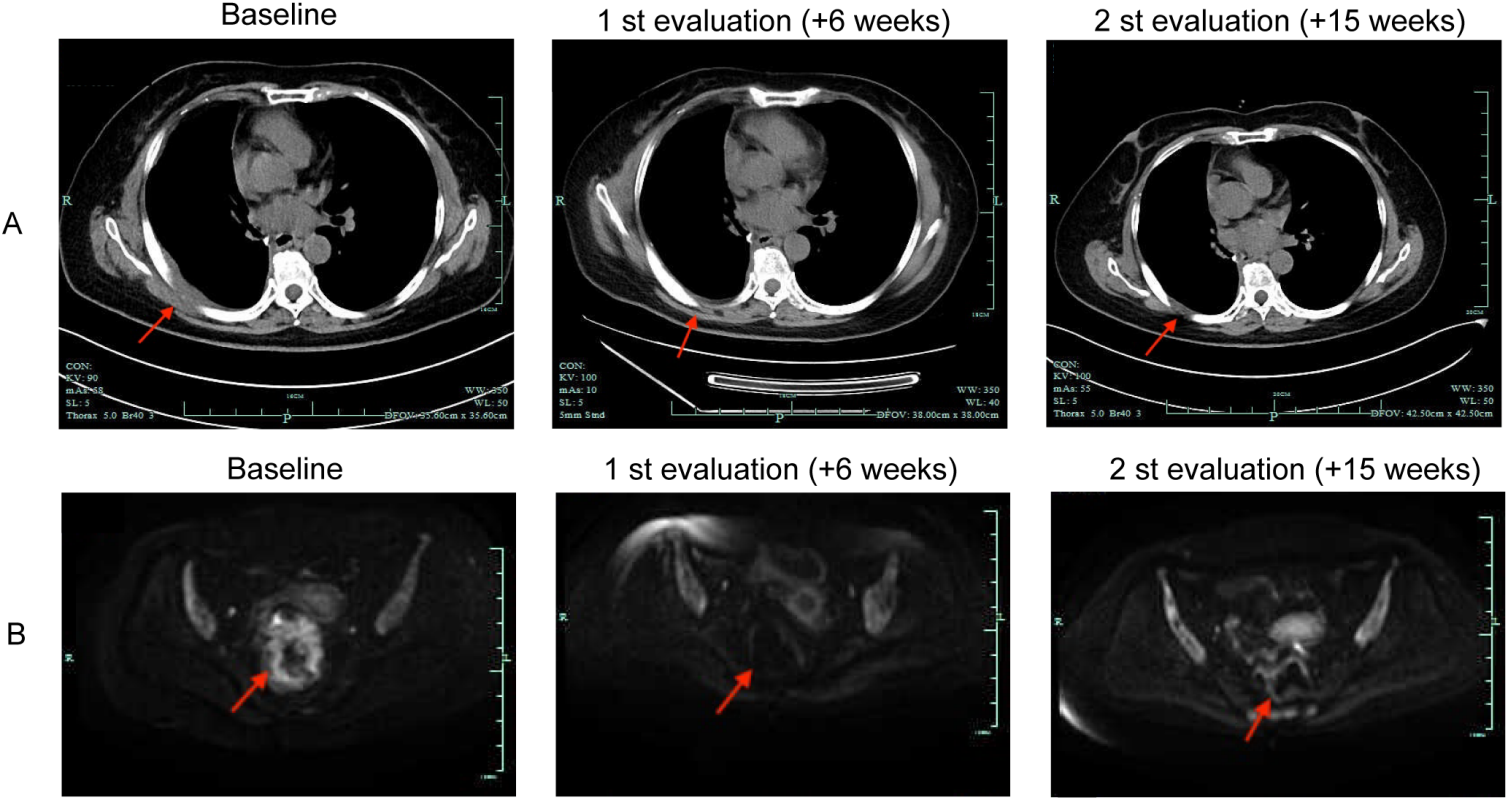
Radiologic images before and after iparomlimab and tuvonralimab. **(A)** Representative chest computed tomography (CT) images. **(B)** Representative pelvic enhanced magnetic resonance imaging (MRI) images.

The patient initiated therapy with 300 mg iparomlimab and tuvonralimab every 3 weeks starting on 8 April 2025. Post-treatment chest CT imaging after two cycles showed that the thickened right pleura has significantly improved and decreased in size (**Figure 4A**). Pelvic enhanced magnetic resonance imaging (MRI) revealed decreased intestinal wall involvement and partial resolution of regional lymphadenopathy (**Figure 4B**). No new lesions or immune-related adverse events were observed, and treatment was continued. After 5 cycles of the treatment, follow-up pelvic MRI indicated further reduction in intestinal wall thickening and local invasion. Colonoscopy with biopsy suggested narrowing at the rectosigmoid junction with no obvious malignant tissue components identified. Chest CT revealed that the paraspinal metastatic lesion remained stable, showing no significant interval change compared to prior imaging. Tumor markers such as CEA and CA125 remained within the normal range. The patient continues to respond to immunotherapy and remains on treatment.

## Discussion

Here, we describe a case of an LS patient with lung adenocarcinoma as the initial presentation. Clinical recognition of LS commonly relies on the Amsterdam criteria II (6) and the modified Bethesda criteria (5). In this patient, a germline MLH1 mutation was identified, and IHC confirmed loss of MLH1 and PMS2 across all tumor sites—lung, colorectal, and the metastatic. A significant family history of early-onset CRC was noted in her brother and daughter, and her son was identified as a mutation carrier, confirming the hereditary nature of the condition.

Double primary tumors refer to two independent primary malignancies in a single patient, occurring simultaneously or sequentially, with distinct histopathological origins and excluding metastasis or recurrence (7). The occurrence of double primary malignancies should raise a red flag for an underlying cancer predisposition syndrome. In particular, the diagnosis of CRC in a young woman with a family history of colon and endometrial cancer is highly suggestive of LS (2). However, this case underscored the diagnostic challenge posed by the presentation of lung adenocarcinoma preceding the onset of a core LS-related cancer, CRC. Lung cancer is the most common cancer and the leading cause of cancer-related mortality worldwide (8). Nevertheless, there is currently no clear evidence linking it to LS. In a large study involving 121 LS families, the incidence of lung cancer in LS patients was found to be similar to that in the general population (9). Similarly, an analysis of 764 MMR gene mutation carriers has indicated increased risks for several extracolonic cancers but not lung adenocarcinoma (10). In addition, a retrospective study of 1179 lung cancer patients found that only 6 patients had pathogenic or likely-pathogenic germline mutations in PMS2, MSH2, or MSH6, and none exhibited MSI or loss of MMR protein expression (11). According to recent literature, the penetrance of extracolonic cancers in MLH1 mutation carriers is well-documented for endometrial, gastric, and urothelial cancers, but robust epidemiological data linking it to lung cancer remains scarce and contentious (12–14). Given the low prevalence of dMMR in lung cancer, MMR IHC or MSI testing is not part of the standard molecular workup for newly diagnosed lung adenocarcinoma. The standard of care focuses on identifying targetable drivers such as EGFR mutations (15). Therefore, the diagnosis in our case was initially missed. It was only the subsequent development of a canonical LS malignancy (CRC) and a metastatic lesion that triggered the hypothesis of an underlying cancer predisposition syndrome. A further challenge arose: whether the lung cancer was a sporadic event in an LS carrier or a manifestation of the syndrome itself. Prior case reports have described lung cancer in patients with known LS without confirming the MMR status of the pulmonary tumors, thereby leaving open the possibility of incidental sporadic cancer in the carriers (16). In our case, the identical dMMR pattern (MLH1/PMS2 loss) in the primary lung, colorectal, and metastatic lesions, corroborated by a germline MLH1 mutation, strongly supports the conclusion that the lung adenocarcinoma was LS-associated rather than sporadic. This case provides compelling evidence that lung adenocarcinoma can be a primary tumor in LS. Notably, retrospective MMR IHC analysis of the archived lung cancer specimen was crucial for characterizing the nature of the lung cancer and establishing the diagnosis of LS after dMMR was identified in the metastatic and colorectal lesions. Therefore, it is strongly recommended that archived tumor tissues from patients with early-onset, multiple, or otherwise suspicious malignancies be evaluated for MMR status when LS is clinically suspected.

The unique biology of dMMR/MSI-H cancers drives their marked sensitivity to immunotherapy (17). The defective DNA mismatch repair system leads to the accumulation of frameshift mutations, which in turn generates a rich landscape of neoantigens (18). These tumor-specific neoantigens are highly immunogenic, fostering the recruitment and infiltration of tumor-reactive T cells into the tumor microenvironment (19). In response, the tumor upregulates immune checkpoint molecules, such as PD-L1 and CTLA-4, as a dominant adaptive resistance mechanism to attenuate the anti-tumor immune response (18). Thus, immune checkpoint inhibitors (ICIs) drive robust T cell responses by recognizing immune evasion, leading to the killing of tumor cells. Although ICIs are recommended for metastatic dMMR/MSI-H cancers regardless of tumor type, clinical evidence supporting their efficacy in rare dMMR malignancies such as lung cancer remains limited (20). This scarcity underscores the critical value of each documented case. Our patient’s clinically significant response to anti-PD-1 therapy (iparomlimab and tuvonralimab) not only aligns with the expected tumor-agnostic principle but also provides confirmatory real-world evidence that this principle holds true even in a tumor type where the dMMR phenotype is exceptionally uncommon. This case, along with previous reports of sustained responses to nivolumab in LS-associated lung cancers (21, 22), reinforces immunotherapy as a pivotal therapeutic strategy for this unique patient subgroup. Emerging evidence suggests that the high clinical efficacy and organ preservation potential of neoadjuvant immunotherapy across dMMR/MSI-H solid tumors, while also solidifying the role of ctDNA as a vital tool for monitoring treatment response (23). Our case corroborates that the presence of dMMR/MSI-H is a superior predictor of response to ICIs than tumor origin, including rare malignancies like lung cancer.

LS is inherited in an autosomal dominant pattern, among which the MLH1 gene is one of the most common predisposing genes, with pathogenic variants conferring a lifetime cancer risk of approximately 80% (1, 24). Accordingly, structured genetic counseling was delivered to the family after the proband’s diagnosis, addressing the inheritance risks of pathogenic variants and associated cancers, as well as the value of predictive testing. The familial aggregation in this kindred, specifically the early-onset CRC in her brother along with the confirmed vertical transmission of an MLH1 mutation to her children, was highly indicative of a hereditary cancer syndrome. Based on the confirmed MLH1 mutation, the intensified surveillance protocol was recommended for at-risk relatives in accordance with international guidelines (25). The clinical utility of this comprehensive approach was decisively demonstrated within this family. The proband’s daughter, upon initiating surveillance following genetic counseling, underwent a timely resection of an early-stage sigmoid adenocarcinoma. The family has initiated an intensified cancer surveillance program, including regular colonoscopies, gastroscopies, and urological evaluations, which is expected to significantly reduce cancer-related morbidity and mortality.

In conclusion, this case highlights the necessity to consider LS in patients with dual primary cancers, especially those involving LS-core tumors and other associated malignancies, as well as in cases with early-onset cancer or a relevant family history. To confirm suspicion, systematic MMR testing via IHC and/or MSI analysis should be performed not only on newly diagnosed tumors but also retrospectively on archived tissue from earlier malignancies. Furthermore, germline genetic testing is essential for definitive diagnosis. Once LS is confirmed, genetic counseling and intensified surveillance should be offered to all at-risk relatives to reduce morbidity and mortality. Moreover, ICIs represents a cornerstone therapy for advanced dMMR/MSI-H tumors, regardless of their origin.

## Data Availability

The datasets presented in this study can be found in online repositories. The names of the repository/repositories and accession number(s) can be found in the article/supplementary material.
